# Supplementary figures and images for: Multi-gene analysis of Symbiodinium dinoflagellates: a perspective on rarity, symbiosis, and evolution
Source: PeerJ. 2014 May 20;2:e394. doi: 10.7717/peerj.394 (PMC4034598; doi:10.7717/peerj.394)

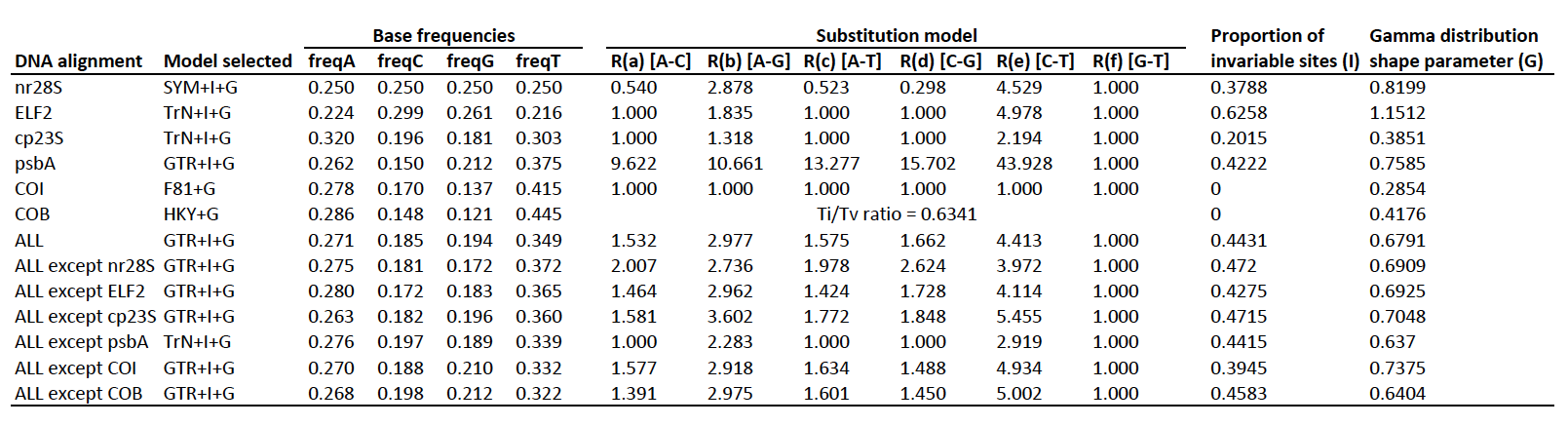

Supplement: Figure S1 [file peerj-02-394-s001.docx]

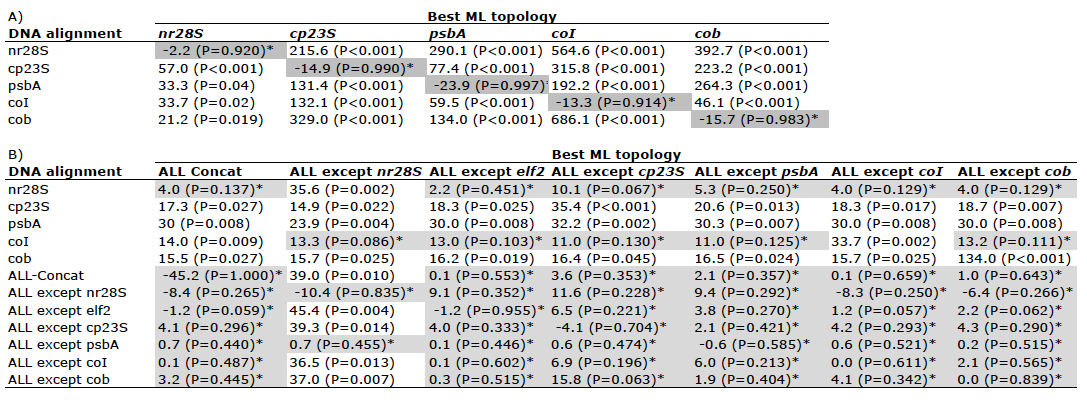

Supplement: Table S2 — For each comparison, Table A and B shows the log likelihood difference and AU test p-value in brackets. *Accepted topologies display a p-value >0.05 (highlighted in grey). (A) Comparisons of single gene DNA alignments to single gene topologies. Elongation Factor 2 (elf2) is missing from these calculations due to missing data (missing sample #27 and #30). (B) Comparisons of single gene and concatenated DNA alignments to the concatenated topologies. elf2 was included in the concatenated alignments, where sample #27 and #30 were coded as missing data. [file peerj-02-394-s002.docx]
